# Ecology in the genomics era of a degraded planet

**DOI:** 10.7554/eLife.02394

**Published:** 2014-02-11

**Authors:** Ian T Baldwin

**Affiliations:** Max Planck Institute for Chemical EcologyJenaGermanybaldwin@ice.mpg.de

**Keywords:** eLife, natural habitat, diversity, population growth

## Abstract

Modern research in ecology draws upon a wide range of different techniques and can offer fresh insights on how to tackle a range of challenges facing the natural world.

Ecology is the study of all the different types of interactions that shape the distribution and abundance of organisms on planet Earth. As such, ecological interactions shape the stream of genetic information that flows forward in time, from generation to generation, and manifests itself as Darwinian evolution. Organisms move this genetic stream forward, with their success being measured by the number of successfully fledged grandchildren they have. However, organisms are not passive conduits for this information to flow through: rather, they are the FedEx employees of the genetic stream, finding new ways to make their deliveries to the next generation more successful by making them more timely, more reliable and more economical. And organisms must be successful and pass along their genetic material, otherwise the stream will evaporate and they will become extinct.

This biological legacy is a vast and unique library, and if we are not careful, we will end up burning it.

By the end of this century, extinction will unfortunately be the likely fate for most of the non-domesticated species on the planet. This is because all organisms require suitable habitats, and *Homo sapiens* currently commands the lion’s share of the best habitats—such as the fresh water, the ice-free land and the net carbon that is fixed by plants—as a consequence of our species having more than seven billion members. While humans have currently attained ‘peak baby’, with the number of children per woman now declining, it is probable that an additional three billion humans will be added to the world’s population by the end of the century as a consequence of the age structure and reproductive potential of the existing population (Rosling, 2012). Under such pressure from our resource-hungry species, it is hard to imagine that many of the other inhabitants of our planet will still be here unless they either directly feed, clothe, house or provide fuel for humans, or if they parasitize humans and the species we have domesticated.

Native habitats have long provided services that we have taken for granted: they purify our water, they stabilize the composition of the atmosphere, and they control floods (Kremen, 2005). They also provide less tangible services—natural habitats are, for example, a source of solace, inspiration, replenishment, comfort and entertainment. Moreover, with on-going advances in genomic tools, biologists can argue more forcefully than ever before that Nature is indeed the Mother of Invention. However, we need to recognize the opportunities that are being opened up by new biological techniques and knowledge, and we must do our best not to squander these opportunities.

Our planet’s biological legacy—including all the different habitats on Earth and all the different species that live in them—is the result of eons of natural selection. In the innumerable as-yet unsequenced genomes of the natural world lie the solutions to the problems that life has faced in the past. This biological legacy is a vast and unique library, and if we are not careful, we will end up burning it. The genomics revolution has fundamentally altered how we can exploit the genetic information in this library: it is as if we have replaced the genetic equivalent of a hard copy encyclopaedia with something like Wikipedia, which means that individual genes can now be moved and manipulated in a variety of innovative ways.

In *eLife* we are striving to publish outstanding articles that illuminate both the peril and the promise of this time in ecological research. Examples of shedding new light on perils include estimating the extinction risks faced by sharks and rays (Dulvy et al., 2014) and sequencing the genome of the pathogen responsible for the Irish potato famine (Yoshida et al., 2013). Other articles have illustrated the awesome power of genomic tools, phylogenetic reconstructions, neurobiological imaging and modelling, as well as old-fashioned morphology and field work, in topics as diverse as the evolution of photosynthesis, the remarkable ability of ants to carry heavy loads, how moths find optimal host plants for their offspring, how plants recruit insects as body guards and how viruses hitchhike on insect vectors to find new hosts. We have also published articles on the evolution of multicellularity in systems as diverse as the choanoflagellates, yeast, protists and bacteria.

In *eLife* we are striving to publish outstanding articles that illuminate both the peril and the promise of this time in ecological research.

As Nature comes more and more under duress, the need to understand and use these triumphs of natural history will increase. The public that funds much of our research is also keen to learn more about what can be done to protect our biological legacy. So, ecologists of the world, send us your best work and together we can make a compelling case for preserving the natural world and all who live in it.
